# Designability of Aromatic Interaction Networks at *E. coli* Bacterioferritin B-Type Channels

**DOI:** 10.3390/molecules22122184

**Published:** 2017-12-08

**Authors:** Yu Zhang, Jinhua Zhou, Maziar S. Ardejani, Xun Li, Fei Wang, Brendan P. Orner

**Affiliations:** 1 College of Chemical Engineering, Jiangsu Provincial Key Lab for the Chemistry and Utilization of Ago-Forest Biomass, Jiangsu Key Lab of Biomass-Based Green Fuels and Chemicals, Nanjing Forestry University, Nanjing 210037, China; 13770559414@163.com (J.Z.); xunlee@njfu.edu.cn (X.L.); hgwf@njfu.edu.cn (F.W.); 2 Division of Chemistry and Biological Chemistry, Nanyang Technological University, 21 Nanyang Link, Singapore 637371, Singapore; sole0001@ntu.edu.sg; 3 Department of Chemistry, King’s College London, London SE1 1DB, UK

**Keywords:** nano-cage, ferritin, B-site interface, self-assembly, protein–protein interaction, protein design

## Abstract

The bacterioferritin from *E. coli* (BFR), a maxi-ferritin made of 24 subunits, has been utilized as a model to study the fundamentals of protein folding and self-assembly. Through structural and computational analyses, two amino acid residues at the B-site interface of BFR were chosen to investigate the role they play in the self-assembly of nano-cage formation, and the possibility of building aromatic interaction networks at B-type protein–protein interfaces. Three mutants were designed, expressed, purified, and characterized using transmission electron microscopy, size exclusion chromatography, native gel electrophoresis, and temperature-dependent circular dichroism spectroscopy. All of the mutants fold into α-helical structures and possess lowered thermostability. The double mutant D132W/N34W was 12 °C less stable than the wild type, and was also the only mutant for which cage-like nanostructures could not be detected in the dried, surface-immobilized conditions of transmission electron microscopy. Two mutants—N34W and D132W/N34W—only formed dimers in solution, while mutant D132W favored the 24-mer even more robustly than the wild type, suggesting that we were successful in designing proteins with enhanced assembly properties. This investigation into the structure of this important class of proteins could help to understand the self-assembly of proteins in general.

## 1. Introduction

Ferritins are functionally important, yet structurally minimal, cage-like protein complexes [1]. The ubiquitous abundance of this protein in nearly all known forms of life reflects their essential role in metabolism [2]. Ferritins are involved in cellular and extracellular homeostasis through the sequestration, storage and release of iron [3]. The self-assembled protein cages of ferritin capture and oxidize excess metabolic iron (Fe(II)) and store it in a mineralized and insoluble form (Fe(III)) inside the protein cage’s central cavity [4]. Despite this vital functional role, ferritin monomers are shorter than the average cellular protein chain length [5]. In all ferritins of known structure, the monomers fold into a four-helix bundle—a relatively simple and well-studied tertiary structure motif. Symmetrical self-assembly of multiple copies of these minimal monomers yields the extended, yet closed, nanostructure of a ferritin cage. Their universal metabolic importance, the simplicity of their hierarchical structure, and their amenability to manipulation with standard molecular biology techniques make ferritins an ideal model system for the development of protein-based functional materials [6,7,8].

Dissecting molecular recognition factors that control the structure-function relationship in ferritins remains an ongoing research endeavor [9]. The crystal structure of a ferritin was first resolved in 1991, revealing that the protein is a 24-meric cage with octahedral symmetry [10]. The exterior cage diameter is ~12 nm, and the interior cavity diameter is ~8 nm. In the octahedral cage, each monomer interacts with six surrounding monomers through three different types of symmetry-related interfaces.

One of the prerequisites for the catalysis of Fe(II) oxidation by ferritin monomers is their higher-order self-assembly [11]. Symmetrical self-assembly of ferritin monomers into a cage-like structure creates channels that guide the Fe(II) ions towards the catalytic centers through the protein shell [12,13]. To the best of our knowledge, the effects of mutations blocking these channels on the self-assembly of ferritins have not been studied.

We and others have shown that, unlike most other ferritins, the native BFR populates two different oligomerization states, 24-mer and dimer, in solution [14,15,16]. The monomeric form of bacterioferritin has never been observed in solution, and there is strong evidence that the dimer is the essential minimal subunit for bacterioferritin self-assembly [14]. Although each B-site interface is made up of an asymmetric arrangement of two dimers, it is symmetrically distributed through the cage structure such that there are 24 quasi-identical B-site interfaces in the BFR nano-cage assembly.

Our studies have demonstrated the possibility of altering the distribution of 24-mer cage and dimer states through single-point mutations [16,17,18]. In one of these studies, we showed that computationally designed symmetric aromatic interactions are able to extend the interaction networks across protein–protein interfaces and shift the oligomerization of BFR towards the cage state [18]. Careful examination of high-order structural information, such as the surface topography of protein–protein interfaces, could facilitate the design of such aromatic interactions. We have shown that, if located in the protein–protein interfaces, native structural pockets provide empty spaces that can be used to build new networks of aromatic interactions in the quaternary structure of proteins [19].

We have previously shown that symmetrically arranged pocket-filling aromatic interactions can enhance the stability and self-assembly of BFR [18,19]. Our previously designed and characterized interactions were symmetric, built in the interfacial pockets around the twofold and threefold axes of symmetry. For this study, we considered potential interfacial pockets at non-symmetric interfaces of BFR. We hypothesized that it would be possible to create non-symmetric networks of aromatic interactions across the B-site interfaces. Here, we present computational, biophysical and structural analyses of mutants that could potentially form aromatic interaction networks at the B-type channels of *E. coli* Bacterioferritin.

## 2. Results and Discussion

### 2.1. Topographic, Bioinformatics and Computational-Aided Design of Aromatic Interactions at the B-Site Interface of BFR

**Figure 1:**
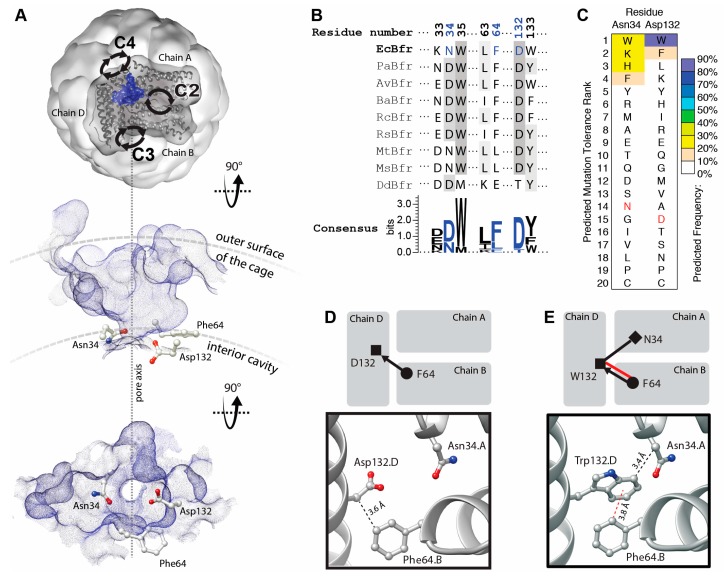
Topographic, bioinformatic and computational analyses of BFR structure facilitate the investigation into designability of aromatic interaction networks at BFR B-site interface. (**A**) Top, topographic analysis of the surface of BFR reveals pockets at the B-site (only one pocket is shown, as a blue surface viewed from outside of the cage). This pocket provides a passage (along the dashed line in A) from the interior cavity of the cage to its outer surface and the bulk solution. Alternative views—perpendicular to the pore axis (middle) and from within the cage (bottom)—show the positioning of three interfacial residues at the constriction of the B-site pore; (**B**) Structure-based multiple sequence alignment of a subset of residues with those positioned at the constriction of BFR B-site pocket colored in blue; (**C**) Computationally predicted occurrence frequency of mutations at Asn34 and Asp132 shows aromatic residues might be energetically favored at these positions. Wild-type residue is shown in red; (**D**) Arrangement of the constriction residues in the wild-type protein shows only one potential VdW interaction between the sidechain of Phe64 and the main chain of Asp132; (**E**) Mutation of Asp132 to Trp creates a new interfacial interaction network, where the Phe64 side chain makes an aromatic interaction with the sidechain and one VdW interaction with the main chain of Trp132. The side chain of Trp132 makes an additional VdW interaction with the sidechain of Asn34.

The B-site protein–protein interfaces of BFR are located midway between the twofold, threefold and fourfold symmetric interfaces (Figure 1A, top). The protein–protein contacts around the B-site interfaces are not symmetric, and therefore the occurrence of mutations is not multiplied by a symmetry factor. Topographic analysis reveals that these interfaces contain a solvent-accessible pocket. Pockets detected at the B-sites encompass a channel that connects the interior cavity to the bulk solution outside of the cage. These channels are termed “B-site pores”, according to Macedo et al. [20]. Three residues—Asn34, Phe64 and Asp132—are involved in the constriction of B-site pores. We hypothesized that positions 34 and 132 could host aromatic residues to create a network of aromatic interactions across the B-site pores through interactions with Phe64 and stabilized the interfaces.

The residues—Asn34 and Asp132—are relatively well conserved compared to those involved in the other interfacial pockets of BFR, and hence may be functionally/structurally more important (Figure 1B). This is consistent with proposals linking these pockets to iron transport [21,22]. Moreover, it has been shown that, in a multiple sequence alignment of homologous proteins, a consensus amino acid residue exhibits a greater-than-average contribution to the stability of the protein in comparison to the non-consensus residues [23]. Thus, this evolutionary conservation suggests that the engineering of aromatic interactions at the B-site pocket may be more challenging than the pockets at the threefold and twofold interfaces [18,19].

However, computationally analysis suggests that some mutations might be tolerated at positions 34 and 132 and that some aromatic residues may even be energetically favored. Mutation to tryptophan is predicted to be the most energetically preferred at position 132 (Figure 1C). Comparison of the WT (Figure 1D) and tryptophan mutant (Figure 1E) interaction networks demonstrates a significant enhancement upon D132W mutation.

### 2.2. Protein Expression and Purification

Plasmids harboring the desired mutations D132W and N34W single- and the related double-mutants were prepared via site-directed mutagenesis using a BFR construct as a template and verified by sequencing. All the mutations were successfully introduced into the expression vector. A series of SDS-PAGE gels was carried out to verify each step of the gene expression and protein purification procedure. The gels demonstrated that all three mutants were expressed as soluble proteins, and purified protein bands were ~19 kD in size (Supplementary Materials Figure S1). Therefore, SDS-PAGE analyses verified that the target proteins were purified to homogeneity.

### 2.3. Size Exclusion Chromatographic Analysis of BFR Assembly in Solution

The solution oligomerization behavior of the mutants with redesigned B-site interfaces was initially analyzed using size exclusion chromatography. This technique separates proteins based on size differences, thereby enabling us to investigate how the designed mutations affect the higher-order structure of the variants.

**Figure 2:**
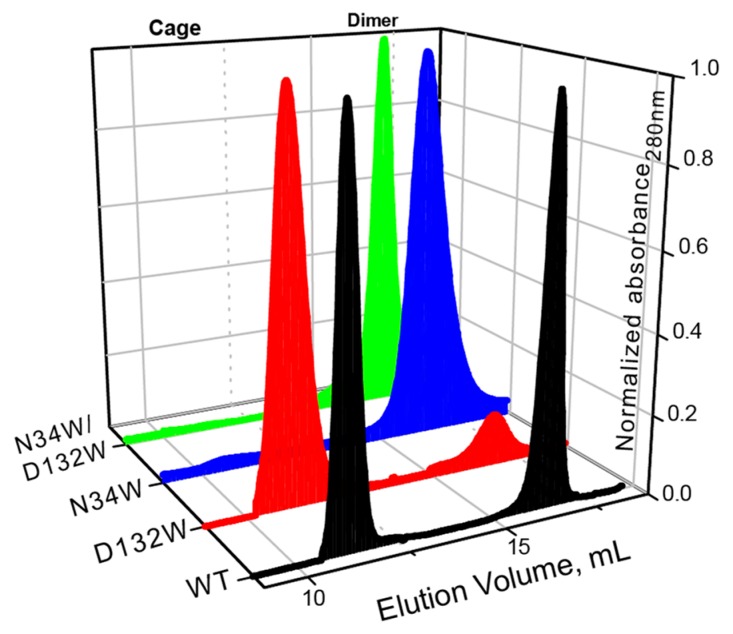
SEC chromatograms of BFR wild type (WT) and three B-site interface mutants, showing that two mutants—N34W and D132W/N34W—form solely dimer, while D132W forms mostly 24-mer cage.

Similar to what we have previously shown, the wild-type BFR elutes as two major peaks with elution volumes corresponding to dimer and 24-mer oligomerization states (Figure 2). In the case of D132W, one of the variants with a redesigned B-site, the dimer peak is smaller than the 24-mer, suggesting that the predicted mutation favors the formation of the higher-order assembly. The ratio of the 24-mer to dimer population in the redesigned mutant is larger than that of wild-type BFR, suggesting that we were successful in designing proteins with enhanced assembly properties. The other two mutants—N34W and D132W/N34W—only formed dimers in solution, indicating that these mutations cripple the ability of protein to form the cage state in solution.

It should be noted that while the redesign of the B-site interface resulted in proteins with relatively subtly enhanced assembly properties, somewhat contrary to the computational analysis, these results were not entirely unexpected. Residues Asp132 and Asn34 are highly conserved among bacterioferritins from different bacteria, implying their importance for functional and structural integrity of this class of protein. Therefore, it is not unreasonable to expect that mutation of these two residues may have an unfavorable (destabilizing) effect on the assembly of cage, even though the free-energy calculations predict otherwise.

While both D132W and N34W mutations happen at the dimer-dimer interface, N34W is also positioned at the dimeric interface. We have previously shown that the dimeric interface is highly sensitive to mutations, to the extent that even isosteric mutations can have detectable crippling effects on the ability of dimers to self-assemble into cages. An explanation for the negative effect of N34W on the self-assembly and thermostability of BFR could be the following: Since N34W happens at a helical region of the dimeric interface and decreases the thermostability of the BFR, and given the fact that tryptophan has a higher helical propensity than asparagine, N34W mutation most probably destabilizes the protein–protein interactions at the dimeric interface. This destabilization could force the dimer to populate a non-assembling, kinetically stable, conformational state incompatible with cage self-assembly.

### 2.4. Oligomerization Preference of Mutants Characterized by Native PAGE

**Figure 3:**
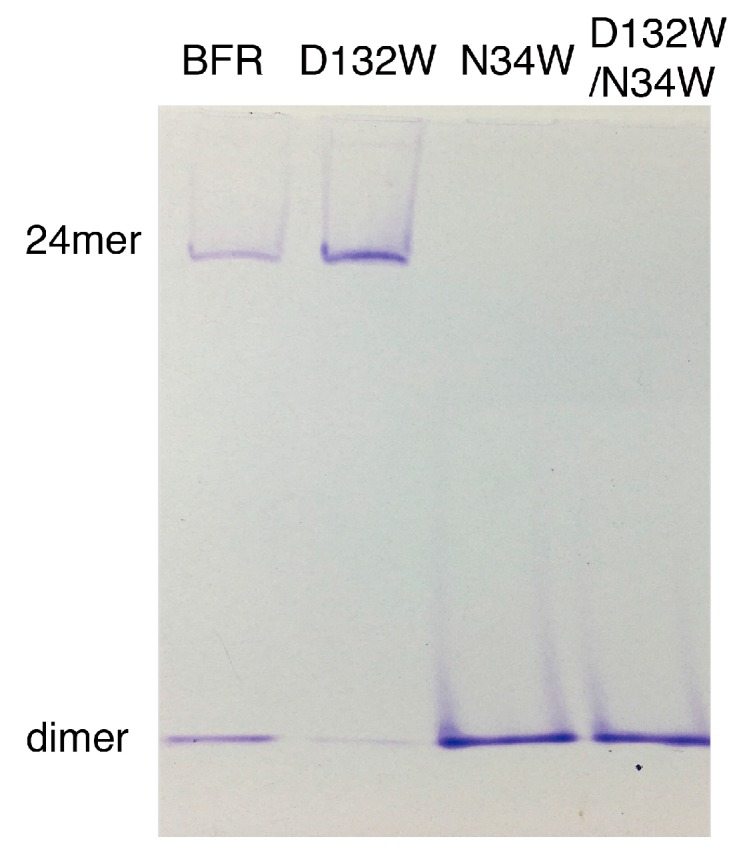
Native PAGE of BFR wild type and three mutants, corresponding to the result of the SEC experiment.

Native PAGE electrophoresis was performed to further confirm the oligomerization preference of the designed mutants. Consistent with the SEC experiment, only the D132W mutant formed two bands, corresponding to the 24-mer and dimer [16,17,18]. All the other mutants generated bands corresponding to wild-type dimer (Figure 3).

### 2.5. TEM Analysis of Protein Assembly

**Figure 4:**
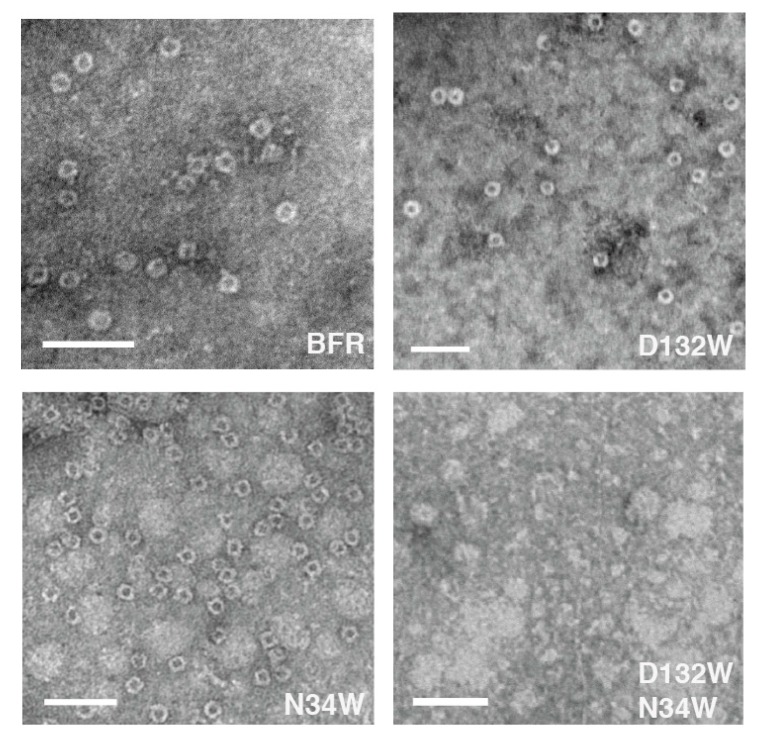
Two of the three BFR-derived mutants can form nano-cages under TEM conditions, the exception being D132W/N34W. Scale bars indicate 50 nm.

The proteins were further analyzed using TEM to determine whether each possessed the ability to assemble into a nano-cage state. Consistent with the SEC, N132W forms cages. In addition, we have noted in the past that some protein cage mutants that are crippled in their ability to form the cage state in solution generate TEM-observable cages [24]. We attributed this observation to the evaporated and surface-immobilized conditions required for TEM, which may be acting to force unfavorable cages together and this is the case with N34W which forms only dimers in solution but cages in TEM conditions. It is interesting to find that, unlike the mutants D132W and N34W, the double mutant D132W/N34W, despite forming dimers in solution, was not able to form cages under TEM forcing conditions (Figure 4). Image analysis of the micrographs indicates that the sizes of the proteins that form cage were comparable to those described in the literature [25] (Table 1).

### 2.6. Analysis of Secondary Structure and Thermal Stability via Temperature-Dependent CD

**Figure 5:**
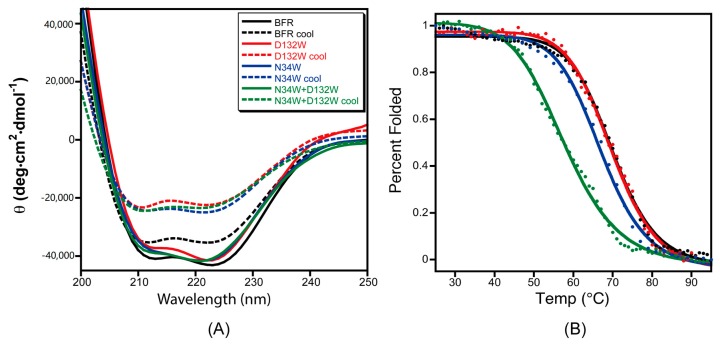
The role mutation plays in the thermal stability and folding reversibility of three mutants at the B-site interface. (**A**) CD spectra of the BFR derivatives before denaturation (solid line) and after slow cooling post-thermal denaturation (dashed lines); (**B**) Thermal transitions of the three mutants at B-site interface as monitored by CD at 222 nm. Data were collected at 1 °C intervals over a temperature range of 25 to 95 °C. The solid lines are the fit and the data points are the experimental data. Data is the average of at least three replicates.

To test the effect of the designed B-site mutations on the thermal stability of BFR, the designed protein variants were subjected to thermal denaturation monitored by CD. The far-UV CD spectra of the proteins in phosphate buffer showed the characteristic double negative minima of α helical secondary structures at 208 nm and 222 nm (Figure 5A). The temperature-dependent CD spectra of the proteins with redesigned B-site interfaces were obtained by scanning the temperature from 25–95 °C. The α-helicity decreased with increasing temperature due to thermal denaturation (Figure 5A). Moreover, unfolding of all of the wild-type BFR and mutant proteins was shown to be partially reversible when the protein samples were slowly cooled from 95 to 25 °C (Figure 5B), making it possible to extract quantitative thermodynamic information on the stability of these proteins from thermal unfolding spectra. Therefore, the negative peak at 222 nm was monitored with respect to temperature to investigate the thermal stability of the proteins.

The thermal unfolding experiments were repeated at least three times for each variant, and the average value of 222 nm CD signal of the repeat spectra were used to construct the melting curves. Assuming a two-state transition, the average CD signals at 222 nm were converted to the fraction folded, and then fit to a simple sigmoid function. By fitting the fraction folded percentage into the two-state sigmoid function, the transition temperatures of thermal unfolding (*T*_m_) were determined (Table 2). The stability of mutant D132W is similar to wild type, while the double mutant D132W/N34W exhibited much lower thermostability than the wild type.

The mutant D132W shows a higher 24-mer to dimer ratio compared to the wild type based on the observations made through size exclusion chromatography and native gel electrophoresis. However, their thermal stability is almost indistinguishable using thermal unfolding monitored by the CD signal at 222 nm. Size exclusion chromatography and native gel electrophoresis characterize the behavior of the proteins at the quaternary structure level, while far-ultraviolet circular dichroism probes the changes in their secondary structure. Although it is expected that stability of quaternary and secondary structures to correlate, there are scenarios where these two levels of structure could be coupled to different degrees. The D132W mutation may stabilize the quaternary structure and favor the cage formation by strengthening the protein–protein interactions at the B-site interfaces. This mutation, however, could have a slight negative effect on the stability of the secondary structure, due to the fact that tryptophan has a lower helix propensity than aspartic acid. These two opposing effects at quaternary- and secondary-structure levels may cancel out each other out, and might be undetectable by thermal unfolding experiments.

## 3. Materials and Methods

### 3.1. Computational Analysis of B-Site Interfaces in BFR

The PDB entry 2vxi of BFR was used for computational analysis. A BFR trimer was created by deleting all except three protein chains centered around a B-site interface (chains A, B and D). Using the FoldX computer program [26], this trimer was first minimized (at 298 K, pH 7.0, 0.05 M ionic strength) in order to generate a reference structure for subsequent energy calculations and structure comparisons. The optimized trimer was then submitted to CASTp [27] for detection and analysis of the structural pockets and Rosetta Backrub [28] for sequence tolerance analysis.

### 3.2. Cloning of the Mutant Genes

Point mutations were introduced into the pET32 Ek/LIC plasmid containing wild-type BFR by site-directed mutagenesis using the primers (Figure S2). The plasmids harboring the respective gene inserts were obtained by miniprep. The sequence of the mutated plasmids was determined by single-path sequencing. The results were aligned with the designed gene sequences using the Blast web tool, which is available online: http://blast.ncbi.nlm.nih.gov/.

### 3.3. Gene Expression and Protein Purification

Plasmids confirmed to have the correct sequence were electroporated into *E. coli* BL21 (DE3) (Novagen, Madison, WI, USA) competent cells. The cells were incubated in 1 mL LB for 1 h, spread on LB plates with carbenicillin (50 μg/mL) and incubated overnight. Single colonies were picked and grown overnight in 3 mL LB with carbenicillin (50 μg/mL). The antibiotic-selected culture was inoculated into 500 mL LB containing 50 μg/mL carbenicillin. The expression culture was induced with IPTG (0.4 mM) until OD600 reached approximately 0.4–0.6, and further incubated (30 °C, 3 h). Bacterial cells were harvested by centrifugation (10,000 rpm, 4 °C, 20 min). The pelleted bacteria were suspended in lysis buffer (50 mM NaH_2_PO_4_, 300 mM NaCl, 10 mM imidazole, pH 8.0) and lysed by sonication. The soluble protein was applied to Ni-NTA resin (QIAGEN) and eluted by affinity tag cleavage following incubation with enterokinase (5 μL, 2.0 μg/mL, 4 °C, 36 h). The protein was concentrated via ultrafiltration (Millipore, Burlington, MA, USA) and further purified by size exclusion chromatography. The final purity of the protein was determined by SDS-PAGE.

### 3.4. Size Exclusion Chromatography

The proteins obtained from affinity purification were equilibrated in gel filtration chromatography buffer (50 mM NaH_2_PO_4_, 150 mM NaCl, pH 7.0) using ultrafiltration tubes (Millipore). The purified proteins were subjected to size exclusion chromatography performed on an ÄKTAFPLCTM (GE Healthcare, Uppsala, Sweden) system using a Superdex 200 10/300 GL gel filtration column pre-equilibrated with running buffer (50 mM NaH_2_PO_4_, 150 mM NaCl, pH 7.0). All the SEC experiments were conducted at 4 °C with a flow rate 0.5 mL/min. The column was previously calibrated using six well-characterized proteins as standards (GE Biosystems Calibration Kit, Eugene, OR, USA). The elution of protein fractions was monitored by measuring UV absorbance at 280 nm. Single peaks corresponding to dimer and 24-mer fractions were collected.

### 3.5. Native PAGE

A 6% gel was run using 15 μg for all the proteins and was stained with Coomassie Blue. Tris-glycine electrophoresis buffer at pH 8.3 was used as gel running buffer.

### 3.6. Transmission Electron Microscopy

A 10 μL solution of purified protein with the concentration of 20 μg/mL was stained using uranyl acetate (1% *w*/*v*). TEM data was obtained using a Joel JEM-1400 transmission electron microscope operating at 100 keV. For each protein, 50 particles were measured.

### 3.7. Temperature-Dependent CD Analysis

The protein solutions were equilibrated in phosphate buffer (50 mM NaH_2_PO_4_, pH 7.2) through extensive dialysis. Protein concentrations were measured by BCA (Novagen) and the solution was diluted (70 μg/mL) with phosphate buffer (50 mM NaH_2_PO_4_, pH 7.2). CD spectra were recorded with a BioLogic MS-500 spectropolarimeter using a 2 mm quartz cell at 222 nm with 1 °C intervals at temperatures ranging from 25 to 95 °C. Once the protein solutions reached 95 °C, they were cooled slowly to 25 °C over 20 min, and the resulting spectra were compared to the spectra obtained at 25 °C before thermal unfolding. At least three replicates were performed.

## 4. Conclusions

The bacterioferritin from *E. coli* was chosen as the basis for investigation of the role of amino acid residues located at the B-site interface. In the X-ray structure of BFR, the B-site is located in a dimer-dimer interface. Stabilization or destabilization of the B-site interface can shift the equilibrium towards cage or dimer state by stabilizing or destabilizing either of these two states. Moreover, based on the working hypothesis of BFR self-assembly, the first step in the cage formation is the association of two dimers through the formation of the B-site at the dimer-dimer interfaces. Therefore, stabilization or destabilization of the B-site interface can also affect the kinetics of cage formation, by affecting the relative rates of formation and lifetimes of dimer and 24-mer during assembly.

Residues involved in B-site pocket are better conserved than those involved in the other interfacial pockets of BFR (Figure 1), and hence may be functionally/structurally more important. This hypothesis is consistent with proposals linking these pockets to iron transport [21]. Moreover, it is well known that consensus amino acid residues exhibit a greater-than-average contribution to the stability of proteins in general compared to non-consensus residues [23]. Thus, this evolutionary conservation of these residues in BFR suggests that the redesign of the B-site pocket-forming residues may be more challenging than those involved in the pockets at the threefold and twofold interfaces [29]. The mutation selected through computational analysis, D132W, favored 24-mer formation over dimer, suggesting that we were successful in designing proteins with enhanced assembly properties by focusing on the B-site.

The functional implications of the results presented here could be twofold. The pore-blocking mutations can stabilize the functionally essential cage form, and at the same time block the passage of ions through B-site channel, which is also necessary for iron mineralization and release. For example, D132W mutation may increase the observed kinetic preference for the cage state, and could therefore potentially increase the ability of the protein to retain the mineralized core due to its more stable cage. However, the mutation could also partially occlude the B-site pores, and hence decrease the mobility of ions through these pores. Further experimental investigation is necessary to unravel the effect of the mutations on the function of *E. coli* bacterioferritin. This investigation into the structure of this important class of proteins can help to understand the self-assembly of proteins in general.

## Figures and Tables

**Table 1:** Average particle diameters of three BFR-derived mutants at B-site interface.

Proteins	Particle Size (nm) ^1^	S.D. (nm)
BFR wild type	12.5	0.8
D132W	12.8	1.0
N34W	12.0	0.7
D132W/N34W	-	-

^1^ For each mutant, at least 50 particles were measured using ImageJ.

**Table 2:** Solution assembly state, melting temperature of BFR-derived mutants at B-site interface.

Proteins	Assembly State	*T*_m_ (°C)	Δ*T*_m_ (°C)
BFR wild type	24-mer & Dimer	69.9	
D132W	24-mer & Dimer	69.7	0.2
N34W	Dimer	67.1	2.8
D132W/N34W	Dimer	57.8	12.1

## Supplementary Materials

Supplementary materials are available online.
