# Supplementary material for: Designability of Aromatic Interaction Networks at E. coli Bacterioferritin B-Type Channels
Source: Molecules. 2017 Dec 8;22(12):2184. doi: 10.3390/molecules22122184 (PMC6149950; doi:10.3390/molecules22122184)
Supplement: Supplementary file 1 [file molecules-22-02184-s001.pdf]

Article

Designability of Aromatic Interaction Networks at *E. coli* Bacterioferritin B-type Channels

Yu Zhang [1]*, Jinhua Zhou [1], Maziar S. Ardejani [2,3], Xun Li [1], Fei Wang [1], Brendan P. Orner [3]*

[1] College of Chemical Engineering, Jiangsu Provincial Key Lab for the Chemistry and Utilization of Ago-forest Biomass, Jiangsu Key Lab of Biomass-Based Green Fuels and Chemicals, Nanjing Forestry University, Nanjing 210037, P. R. China; yuzhang@njfu.edu.cn (Y.Z.); 13770559414@163.com (J.Z.); xunlee@njfu.edu.cn (X.L.); hgwf@njfu.edu.cn (F.W.).

[2] Division of Chemistry and Biological Chemistry, Nanyang Technological University, 21 Nanyang Link, Singapore 637371; sole0001@ntu.edu.sg (M.S.A.)

[3] King's College London, Department of Chemistry, London, United Kingdom; Brendan.patrick.orner@gmail.com (B.P.O.)

* Correspondence: yuzhang@njfu.edu.cn; Brendan.patrick.orner@gmail.com; Tel.: +86-25-85427635; Fax: +86-25-85427649

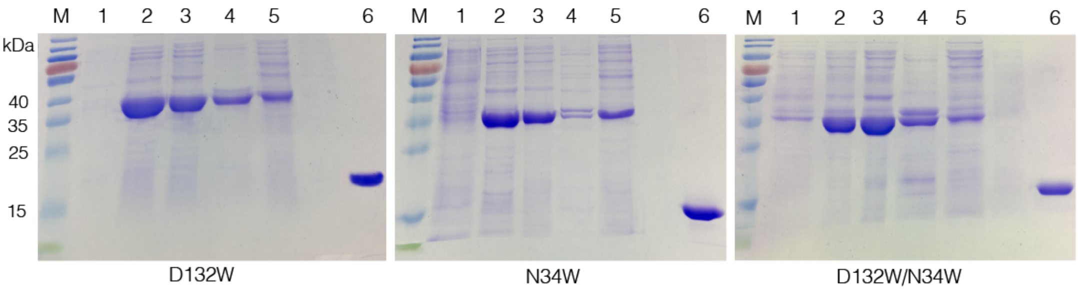

Figure S1 Electrophoretic monitoring of the purification of the BFR-derived proteins. Lane M: ladder. Lane 1: before induction. Lane 2: post induction. Lane 3: soluble fraction. Lane 4: insoluble fraction. Lane 5: flow through. Lane 6: purified protein after cleavage.

D132W

D132W-F（5’-3’）： *TGG*TGGCTGGAAACCGAACTG

D132W-R（5’-3’）： GATATGACCTTCTTCATCACGC

N34W

N34W-F（5’-3’）： CCA*CCA*TTTGAACATACGTGCATGCAG

N34W-R（5’-3’）： CGTATGTTCAAA*TGG*TGGGGTCTGAAACGTCTG

D132W/N34W was performed on the basis of plasmid D132W.

Figure S2. Primers used in QuikChange mutagenesis for D132W, N34W and D132W/N34W using the BFR construct as a template.

Sequencing Results

The sequencing results were aligned with the BFR gene using BLAST (NCBI). The mutant codon is highlighted in red.

D132W: (D-GAT; W-TGG)

Query is BFR native, Sbjct is the sequencing result.

```
Query 1    .....ATGAAAGGTGATACCAAAGTGATCAACTACCTGAACAAACTGCTGGGTAACGAAC
                |||||||||||||||||||||||||||||||||||||||||||||||||||||||
Sbjct 1    ACAAGATGAAAGGTGATACCAAAGTGATCAACTACCTGAACAAACTGCTGGGTAACGAAC

Query 56   TGGTGGCAATCAACCAGTACTTCCTGCATGCACGTATGTTCAAAAACTGGGGTCTGAAAC
           ||||||||||||||||||||||||||||||||||||||||||||||||||||||||||||
Sbjct 61   TGGTGGCAATCAACCAGTACTTCCTGCATGCACGTATGTTCAAAAACTGGGGTCTGAAAC

Query 116  GTCTGAACGATGTGGAATACCATGAAAGCATCGATGAAATGAAACATGCAGATCGTTACA
           ||||||||||||||||||||||||||||||||||||||||||||||||||||||||||||
Sbjct 121  GTCTGAACGATGTGGAATACCATGAAAGCATCGATGAAATGAAACATGCAGATCGTTACA

Query 176  TCGAACGTATCCTGTTCCTGGAAGGTCTGCCGAACCTGCAGGATCTGGGTAAACTGAACA
           ||||||||||||||||||||||||||||||||||||||||||||||||||||||||||||
Sbjct 181  TCGAACGTATCCTGTTCCTGGAAGGTCTGCCGAACCTGCAGGATCTGGGTAAACTGAACA

Query 236  TCGGTGAAGATGTGGAAGAAATGCTGCGTAGCGATCTGGCACTGGAACTGGATGGTGCAA
           ||||||||||||||||||||||||||||||||||||||||||||||||||||||||||||
Sbjct 241  TCGGTGAAGATGTGGAAGAAATGCTGCGTAGCGATCTGGCACTGGAACTGGATGGTGCAA

Query 296  AAAACCTGCGTGAAGCAATCGGTTACGCAGATAGCGTGCATGATTACGTGAGCCGTGATA
           ||||||||||||||||||||||||||||||||||||||||||||||||||||||||||||
Sbjct 301  AAAACCTGCGTGAAGCAATCGGTTACGCAGATAGCGTGCATGATTACGTGAGCCGTGATA

Query 356  TGATGATCGAAATCCTGCGTGATGAAGAAGGTCATATCGATTGGCTGGAAACCGAACTGG
           ||||||||||||||||||||||||||||||||||||||   |||||||||||||||||||
Sbjct 361  TGATGATCGAAATCCTGCGTGATGAAGAAGGTCATATCTGGTGGCTGGAAACCGAACTGG

Query 416  ATCTGATCCAGAAAATGGGTCTGCAGAACTACCTGCAGGCACAGATCCGTGAAGAAGGT
           |||||||||||||||||||||||||||||||||||||||||||||||||||||||||||
Sbjct 421  ATCTGATCCAGAAAATGGGTCTGCAGAACTACCTGCAGGCACAGATCCGTGAAGAAGGTT
```

N34W: (N-AAC; W-TGG)

Query is BFR native, Sbjct is the sequencing result.

```
Query 1   ..............ATGAAAGGTGATACCAAAGTGATCAACTACCTGAACAAACTGCTGG
                        ||||||||||||||||||||||||||||||||||||||||||||||
Sbjct 1   ATGACGACGACAAGATGAAAGGTGATACCAAAGTGATCAACTACCTGAACAAACTGCTGG
```

```
query 47  GTAACGAACTGGTGGCAATCAACCAGTACTTCCTGCATGCACGTATGTTCAAAAACTGGG
          |||||||||||||||||||||||||||||||||||||||||||||||||||||   ||||
sbjct 61  GTAACGAACTGGTGGCAATCAACCAGTACTTCCTGCATGCACGTATGTTCAAATGGTGGG

query 107 GTCTGAAACGTCTGAACGATGTGGAATACCATGAAAGCATCGATGAAATGAAACATGCAG
          ||||||||||||||||||||||||||||||||||||||||||||||||||||||||||||
sbjct 121 GTCTGAAACGTCTGAACGATGTGGAATACCATGAAAGCATCGATGAAATGAAACATGCAG

query 167 ATCGTTACATCGAACGTATCCTGTTCCTGGAAGGTCTGCCGAACCTGCAGGATCTGGGTA
          ||||||||||||||||||||||||||||||||||||||||||||||||||||||||||||
sbjct 181 ATCGTTACATCGAACGTATCCTGTTCCTGGAAGGTCTGCCGAACCTGCAGGATCTGGGTA

query 227 AACTGAACATCGGTGAAGATGTGGAAGAAATGCTGCGTAGCGATCTGGCACTGGAACTGG
          ||||||||||||||||||||||||||||||||||||||||||||||||||||||||||||
sbjct 241 AACTGAACATCGGTGAAGATGTGGAAGAAATGCTGCGTAGCGATCTGGCACTGGAACTGG

query 287 ATGGTGCAAAAAACCTGCGTGAAGCAATCGGTTACGCAGATAGCGTGCATGATTACGTGA
          ||||||||||||||||||||||||||||||||||||||||||||||||||||||||||||
sbjct 301 ATGGTGCAAAAAACCTGCGTGAAGCAATCGGTTACGCAGATAGCGTGCATGATTACGTGA

query 347 GCCGTGATATGATGATCGAAATCCTGCGTGATGAAGAAGGTCATATCGATTGGCTGGAAA
          ||||||||||||||||||||||||||||||||||||||||||||||||||||||||||||
sbjct 361 GCCGTGATATGATGATCGAAATCCTGCGTGATGAAGAAGGTCATATCGATTGGCTGGAAA

query 407 CCGAACTGGATCTGATCCAGAAAATGGGTCTGCAGAACTACCTGCAGGCACAGATCCGTG
          ||||||||||||||||||||||||||||||||||||||||||||||||||||||||||||
sbjct 421 CCGAACTGGATCTGATCCAGAAAATGGGTCTGCAGAACTACCTGCAGGCACAGATCCGTG

query 467 AAGAAGGT
          ||||||||
sbjct 481 AAGAAGGT
```

D132W-N34W： (D-GAT; N-AAC; W-TGG)

Query is BFR native, Sbjct is the sequencing result.

```
Query 1   ..ATGAAAGGTGATACCAAAGTGATCAACTACCTGAACAAACTGCTGGGTAACGAACTGG
            ||||||||||||||||||||||||||||||||||||||||||||||||||||||||||
Sbjct 1   AGATGAAAGGTGATACCAAAGTGATCAACTACCTGAACAAACTGCTGGGTAACGAACTGG

Query 59  TGGCAATCAACCAGTACTTCCTGCATGCACGTATGTTCAAAAACTGGGGTCTGAAACGTC
            |||||||||||||||||||||||||||||||||||||||||   ||||||||||||||||
Sbjct 61  TGGCAATCAACCAGTACTTCCTGCATGCACGTATGTTCAAATGGTGGGGTCTGAAACGTC
```

```
Query  119TGAACGATGTGGAATACCATGAAAGCATCGATGAAATGAAACATGCAGATCGTTACATCG
          ||||||||||||||||||||||||||||||||||||||||||||||||||||||||||||
Sbjct  121 TGAACGATGTGGAATACCATGAAAGCATCGATGAAATGAAACATGCAGATCGTTACATCG

Query  179AACGTATCCTGTTCCTGGAAGGTCTGCCGAACCTGCAGGATCTGGGTAAACTGAACATCG
          ||||||||||||||||||||||||||||||||||||||||||||||||||||||||||||
Sbjct  181 AACGTATCCTGTTCCTGGAAGGTCTGCCGAACCTGCAGGATCTGGGTAAACTGAACATCG

Query  239GTGAAGATGTGGAAGAAATGCTGCGTAGCGATCTGGCACTGGAACTGGATGGTGCAAAAA
          ||||||||||||||||||||||||||||||||||||||||||||||||||||||||||||
Sbjct  241 GTGAAGATGTGGAAGAAATGCTGCGTAGCGATCTGGCACTGGAACTGGATGGTGCAAAAA

Query  299ACCTGCGTGAAGCAATCGGTTACGCAGATAGCGTGCATGATTACGTGAGCCGTGATATGA
          ||||||||||||||||||||||||||||||||||||||||||||||||||||||||||||
Sbjct  301 ACCTGCGTGAAGCAATCGGTTACGCAGATAGCGTGCATGATTACGTGAGCCGTGATATGA

Query  359TGATCGAAATCCTGCGTGATGAAGAAGGTCATATCGATTGGCTGGAAACCGAACTGGATC
          |||||||||||||||||||||||||||||||||||   ||||||||||||||||||||||
Sbjct  361 TGATCGAAATCCTGCGTGATGAAGAAGGTCATATCTGGTGGCTGGAAACCGAACTGGATC

Query  419 TGATCCAGAAAATGGGTCTGCAGAACTACCTGCAGGCACAGATCCGTGAAGAAGGT
           ||||||||||||||||||||||||||||||||||||||||||||||||||||||||
Sbjct   421 TGATCCAGAAAATGGGTCTGCAGAACTACCTGCAGGCACAGATCCGTGAAGAAGGT
```
